# Cases of Acute Hydrocephalus Treated by the Iodide of Potassium

**Published:** 1842-10-29

**Authors:** 


### ANALECTA.

*Cases of Acute Hydrocephalus treated by the Iodide of Potassium.*—Dr. FLUDER, of Lymington, reports, in the London Medical Gazette, September 30th, some interesting cases of acute hydrocephalus cured, in the last stage, by the iodide of potassium. He says:—

"Seventeen or eighteen months ago, two cases of acute hydrocephalus, in children about a year old, were in progress of treatment, by my partner, Mr. Chinery. He had had recourse to bleeding with moderation—also to mercurials and other remedies proportioned to the strength and powers of the little patients, and the whole of the treatment had been conducted on rational principles. The gums had been freely scarified, though there was no evidence that the cerebral irritation was immediately connected with dentition. One of the children had rather a large head, and might have been the subject of some chronic disease on which inflammatory action had supervened, and the child was certainly of a scrofulous diathesis; not so with the other. It was previously healthy, born of healthy parents, had gone through the usual symptoms described by authors as appertaining to acute hydrocephalus, and had been treated accordingly; but despite of all measures was apparently in a dying condition. The mother, though fondly attached to her child, was exceedingly averse, conceiving its entire inutility, to making any further trial of medicine; and I should say that medicine of any kind was only got into the stomach by putting it far back on the tongue, and depressing the organ till deglutition was unavoidably accomplished. This child, in addition to strabismus, laboured respiration, convulsions, and other symptoms, supposed by some to be indicative of effusion, had for several days together complete opisthotonos, the body being stiffly arched from the occiput to the ossa calcium. Both children were occasionally convulsed, comatose, and miserable for days together, and in this condition I suggested a trial of the iodide of potassium, in half grain-doses, every two or three hours. The only visible effect of this, in connection with the rapid improvement of both children, was considerable diuresis, and in one, much saliva dribbled from the mouth. Both recovered perfectly and speedily, and are living and healthy at this time.

An equally well-marked case has occurred to me more recently. On the 10th of May last, I visited the infant child of a small farmer, on the borders of the New Forest. The child, about fourteen months old, robust, and previously healthy, exhibited the usual symptoms of pyrexia. Considering that dentition might be the source of irritation I lanced the gums freely, and as the bowels were costive, directed a purge of calomel and jalap. The next day the child had become more feverish, occasionally screaming, and exhibiting annoyance at light. The tongue and breath were foul; the pulse quick and varying; the countenance flushed; and the scalp very hot. Leeches were applied to the temples and a cold lotion to the head, and in the evening, the respiration being laboured, and the more especially as crepitation was observed over the right lung, four leeches were applied below the clavicle, a nauseating mixture was prescribed, and the calomel purge repeated. After this the breathing was somewhat better, but the febrile symptoms continued. On the 12th and 13th, the child remained in very much the same state, but nearly insensible to objects around it. The bowels obstinately costive; the evacuations that were sparingly obtained from them by different medicines were of a dark colour. A blister was applied to the nape of the neck, calomel was administered every four or five hours, and a mixture of nitre and acetate of ammonia.

On the 14th, extreme stupor seemed to overwhelm the child; the pulse, which throughout had been very quick, became more irregular and indistinct, and a clammy sweat overspread the body.

On the 15th the pupils were dilated and immoveable, and insensible to light. There was complete paralysis of the right side, while the limbs of the left were in constant tremulous motion, the hand being frequently drawn to the head with an undulatory automatic movement. This state of things continued to the 18th, with occasional convulsions and almost entire insensibility. Wine had been administered without benefit, and I do not know how it was that I did not prescribe the iodide of potassium a day or two earlier, for the effects of that medicine, in the two cases before alluded to, had made a considerable impression on my mind at the time. Without much expectation of doing good in this case, I prescribed the medicine as before, and with precisely the same results; not indeed instantaneous, but as an observant grandmother remarked, it appeared to improve with each dose of the medicine; the only appreciable effects being a speedy diuresis and dribbling of saliva."

